# The Importance of Considering Alexithymia during Initial Stages of Intimate Partner Violence Interventions to Design Adjuvant Treatments

**DOI:** 10.3390/ijerph16193695

**Published:** 2019-10-01

**Authors:** Ángel Romero Martínez, Marisol Lila, Luis Moya-Albiol

**Affiliations:** 1 Department of Psychobiology, University of Valencia, 46010 Valencia, Spain; Luis.Moya@uv.es; 2 Department of Social Psychology, University of Valencia, 46010 Valencia, Spain; Marisol.Lila@uv.es

**Keywords:** alexithymia, dropout, drug misuse, intimate partner violence against women, reoffending

## Abstract

Empirical evidence supports that individuals with alexithymia might be prone to certain types of violence, such as intimate partner violence against women (IPVAW). Moreover, considering that alexithymia is directly involved in behavior regulation, problems due to identifying and regulating emotional states might be postulated as responsible, at least in part, for the success of psychotherapeutic treatments designed for decreasing the future risk of reoffending. Therefore, we assessed whether alexithymia was a good predictor of the discontinuation of treatment (dropout) and the risk of recidivism during the initial stages of intervention in a sample of men convicted of IPVAW perpetration (*n* = 118), while controlling for potential confounding variables (i.e., socio-demographic characteristics, impulsivity, and drug misuse). Our data demonstrate that high alexithymic traits lead to dropout and a high risk of recidivism during the initial stages of treatment, remaining significant even after including potential confounding variables. Even though impulsivity and drug misuse increase the amount of explained variance, none of them moderates the association of alexithymia with dropout and/or reoffending. It should be noted that this study identified alexithymia as a direct modulator of violence due to its effects on discontinuing IPVAW treatment during initial stages. Therefore, as we gain knowledge analyzing the relationships between previously mentioned factors, it could be applied to develop valid screening methods along with strengthening current rehabilitation programs and designing adjuvant treatments to increase their effectiveness.

## 1. Introduction

A significant proportion of individuals present serious difficulties identifying, describing, and/or expressing/verbalizing their feelings as well as trouble differentiating between body sensations and feelings during emotionally arousing experiences. Moreover, these individuals often present a restricted imagination and a tendency to experience hostility when they cope with stress. All these symptoms have been defined as alexithymia [1,2,3,4,5], which has awakened scientific interest during the last years in regard to understanding its role in individuals who present difficulties regulating their behavior, including violent behavior.

Empirical evidence supports that the existence of alexithymia might be related to the perpetration of intimate partner violence against women (IPVAW) [6,7], specifically non-premeditated or impulsive violence [8] and psychological IPVAW [6]. These conclusions were further confirmed by a recent published manuscript. In this sense, alexithymic traits and their relation to several cognitive and empathic variables in IPVAW perpetrators were analyzed. The authors concluded that alexithymic traits were associated with dysexecutive symptoms, high impulsivity, and poor empathic skills [9]. Moreover, these symptoms could be worse if individuals present other risk factors for violence, such as drug misuse [10,11]. This might explain the tendency of these individuals to experience violent outbursts when they cope with stress. In other words, the restricted ability to connect and verbalize their inner states, in combination with an impaired ability to process external cues, high impulsivity traits, as the presence of hostile cognitive schemas, serious difficulties regulating emotional expression, and drug misuse support the emergence of violence [9,10]. 

In regard to alexithymia’s impact on emotional and behavioral regulation, these difficulties with acknowledging, recognizing, and regulating emotional states might be postulated as directly responsible, at least in part, for the success of psychotherapeutic treatments [10,12,13,14]. Thus, it makes sense to conclude that the presence of alexithymia might entail an overwhelming sensation caused by the intervention’s content, which could seriously affect patients’ behavioral and emotional regulation improvements after interventions designed for them. Unfortunately, this has yet to be analyzed in IPVAW perpetrators. Therefore, it would be necessary to assess the impact of alexithymia during the intervention’s initial stages in order to assess its impacts on IPVAW perpetrators’ discontinuation of psychotherapeutic treatment (dropout) and risk of reoffending. 

Considering all the above, the first objective of this study was to assess whether or not alexithymia predicts the dropout and the risk of recidivism during initial stages of intervention in a carefully selected sample of men convicted of IPVAW perpetration, while controlling for potential confounding variables (i.e., socio-demographic characteristics, personality traits, and drug misuse). We expected to find that alexithymia would significantly predict the dropout and the initial risk of recidivism in consonance with previous conclusions in this field of research [6,7,9,10]. Furthermore, we also expected that these results would remain significant when including potential confounding variables. The second objective was to explore whether impulsivity and drug misuse moderates the association between alexithymia and the previously mentioned variables (dropout and risk of reoffending). Following the conclusions of previous research [9,15,16,17], we expected to find a solid link between alexithymia, dropout, and the risk of recidivism in IPVAW perpetrators with the highest levels of impulsivity and drug misuse. 

## 2. Method

### 2.1. Participants

After the initial screening, the final sample consisted of 118 IPVAW perpetrators. Participants for this study were recruited from the CONTEXTO psycho-educational and community-based treatment program (mandatory in Spain for male abusers). It should be noted that IPVAW perpetrators who were sentenced to less than two years in prison (from approximately 6 to a maximum of 24 months) and had no previous criminal record received a suspended sentence on the condition that they attend this intervention program [18]. For our study, the following participants were included: men over 18 years of age, no severe substance use disorders, cognitive impairments (i.e., brain damage, current strokes with severe cognitive impairments, degenerative disorders), and/or mental problems (i.e., schizophrenia, psychosis). In this sense, two researchers trained in IPVAW interventions conducted two separate interviews with participants in order to detect psychopathology and personality disorders. The content of this qualitative interview was based on the psychopathological dimensions evaluated by the Symptom Checklist-90-Revised (SCL-90-R) and the Millon Clinical Multiaxial Inventory (MCMI-III). The degree of inter-rater agreement (Cohen’s kappa) on the different symptoms and disorders was higher than 0.76 for all assessed areas. Once both researchers detected no severe mental or personality disorders, candidates became eligible to participate in the intervention program. Furthermore, these interviews were completed by several self-reports. It should be noted that participants completed the SLC-90-R and MCMI-III questionnaires and were included in our study if they scored below the mean for their age on each dimension of these self-reports.

Participants became a part of this study after being informed about its nature. After agreeing to participate by signing the informed consent, they were included in our study. This was conducted in accordance with the Declaration of Helsinki and approved by the University of Valencia Ethics Committee (H1348835571691).

### 2.2. Procedure 

The data for our study [18,19,20,21] was collected during the initial stages of the CONTEXTO program. Participants were required to attend two sessions in the psychology laboratories. Initially, they were interviewed to exclude those individuals with serious difficulties, such as physical or mental disorders. Additionally, the existence of drug misuse was analyzed with an interview based on DSM-5 criteria for substance use disorders. Finally, a battery of self-reports was registered during a second session.

#### 2.2.1. Self-Reports (Psychological Variables)

Alexithymia was studied with the Toronto Scale of 20 Elements (TAS-20) [22], correctly adapted and validated in Spanish [23]. The scale contains 20 Likert-type items rated on a 6-point scale (from 0 to 5) (i.e., I am often confused about what emotion I am feeling; It is difficult for me to find the right words for my feelings.) A total score higher than 61 indicates the presence of alexithymia. Cronbach’s alpha was 0.86.

To measure impulsivity, we employed the Plutchik Impulsivity Scale [24], conveniently adapted in Spanish [25]. This test consists of 15 items, with responses ranging from 1 (never) to 4 (almost always). A total score higher than 26 indicates the presence of impulsivity. Cronbach’s alpha was 0.85.

#### 2.2.2. Drug Misuse Assessment

We registered the frequency and amount of drug use (i.e., alcohol, marijuana, cocaine). Participants were classified as consumers if they presented an elevated weekly consumption, but they were categorized as non-consumers if they did not consume any drugs during the last 6 months or if they registered occasional and non-sustained consumption during this period. Moreover, the adapted Spanish version of the Alcohol Use Disorders Identification Test (AUDIT) [26] was employed to check for alcohol use. This test consists of 10 items, which range from 0 (never) to 4 (daily). The cut-off score is 8 for discriminating non-risky from risky drinkers (i.e., heavy and sustained alcohol consumption). Cronbach alpha was 0.88.

#### 2.2.3. Dropout and Risk of Recidivism

The number of treatment sessions received (intervention dose) as well as the discontinuation of the treatment program (yes vs. no) were registered by researchers of the CONTEXTO program. Both variables were employed as dependent variables for dropout. One of them was considered a continuous variable (number of sessions) and the other one as qualitative.

The risk of recidivism was assessed with the conveniently adapted version of the Spousal Assault Risk Assessment Guide (SARA) [27]. This test consists of 20 items ranked with a 3-point scale (0 = absence; 1 = possibly present; 2 = presence), which assesses violence risk in general and IPVAW. A total risk score is calculated as the sum of scores on the 20 items.

### 2.3. Data Analysis 

To analyze the direct association between alexithymia and the dependent variables (number of treatment sessions, dropout, and SARA), we performed linear regression and logistic regression analyses. In order to assess the impulsivity and drug misuse moderation, we followed the methodology of Aiken and West [28], and Dawson and Richter [29] to plot the interaction effects. In this regard, we previously assessed if all variables were significantly related by correlation. As some potential moderators are quantitative (impulsivity and AUDIT score), it was necessary to dummy code these variables before forming the product term that represented the interaction. In step 1, we included the alexithymia as an independent variable (IV 1). In step 2, we added impulsivity, dummy coded, (IV 2), or drug misuse (IV 2) as predictors. Finally, in step 3, we added the interaction term “alexithymia*impulsivity dummy coded” or “alexithymia*drug misuse” to investigate whether the relationship between alexithymia and dropout or risk of reoffending was moderated by the impulsivity or the drug misuse of the participants. Due to the large number of comparisons, a Bonferroni adjustment for multiple comparisons was performed, setting the significance level at 0.001. 

Data analyses were carried out using IBM SPSS Statistics for Windows, Version 24.0 (Armonk, NY, USA). 

## 3. Results

IPVAW perpetrators’ mean age was 41.47 years old (SD = 12.46). Most of them were Spanish, divorced or single, presented a primary educational level and were actively working (see Table 1). Regarding their drug misuse, IPVAW perpetrators’ alcohol consumption was under the risk score (less than an average score of 8), many of them did not present an active use of cannabis and/or cocaine. Lastly, only a small percentage of participants dropped out of the intervention and their risk of reoffending during the initial stages of treatment was low according to SARA interpretation (maximum score of 40).

**Self-reported alexithymic traits as predictors of intervention dose, dropout, and risk of recidivism.** Specifically, the self-reported alexithymic traits predicted 0.116 of the variance in intervention dose (*β* = −0.352, *p* < 0.001; *F* = 16.24, *p* = 0.001, 95% CI = −0.36 to −0.12), 0.121 of the variance in official reoffending (*β* = 0.044, *p* < 0.001; Exp(B) = 1.044, *p* = 0.001, 95% CI = 1.014 to 1.076) and 0.133 in risk of recidivism (*β* = 0.374, *p* < 0.001; *F* = 18.59, *p* = 0.001, 95% CI = 0.08 to 0.20). After including the participant’s age, educational level, drug misuse (alcohol, marijuana, and cocaine), and self-reported impulsivity, the prediction of self-reported alexithymic traits remained significant for intervention dose (*β* = −0.318, *p* < 0.001, 95% CI = −0.36 to −0.09), dropout (Exp(B) = 0.043, *p* < 0.01, 95% CI = 1.01 to 1.08), and risk of recidivism (*β* = 0.279, *p* < 0.001, 95% CI = 0.03 to 0.18). 

Afterward, quantitative variables (alcohol and self-reported impulsivity), which previously were included as covariates, were dummy coded in order to check whether they moderated the association between alexithymia and intervention dose, dropout, and risk of recidivism. Most of these variables were significantly associated with alexithymia and the dependent variables (see Table 2). Moreover, after being included in step 2, the amount of explained variance significantly increased (*p* < 0.001) but the interaction of alexithymia with impulsivity and drug misuse (independent variables) did not increase the amount of explained variance (Step 3, *p* > 0.05). Hence, we cannot assume the moderation between these variables.

## 4. Discussion

In IPVAW, perpetrators’ high alexithymic traits partially explain the dropout and high risk of recidivism during initial stages of treatment. These predictions were still significant even after including confounding variables, such as socio-demographic characteristics, impulsivity, and drug misuse. Even though impulsivity and drug misuse also entailed high alexithymia, low number of treatment sessions received, high dropout rates, and high risk of reoffending rates, these variables did not moderate the association between the previously mentioned parameters. 

As expected, alexithymia significantly predicted dropout and risk of reoffending. Additionally, it is necessary to keep in mind that our data also revealed that the IPVAW perpetrators presented an average score which was higher than the cut-off score ( > 61) on TAS-20 which indicated the existence of a marked presence of alexithymia in this group. These results were in line with previous studies in this field of research [6,7,9,10,11]. We also demonstrated the robust association of alexithymia with dropout and risk of reoffending. In fact, after controlling for confounding variables, the relationships remained significant. Furthermore, this result was reinforced by the fact that we statistically controlled for multiple comparisons, thus we cannot explain our results by type I error. However, it is important to acknowledge that the percentage of variance predicted for alexithymia is relatively low. Hence, alexithymia itself is not enough to completely predict dropout and reoffending. In this sense, several studies have stated the importance of considering several subtypes of IPVAW perpetrators with different cognitive characteristics and, in turn, therapeutic needs [19,30,31,32,33]. Our data reinforces this conclusion and strengthens the need to analyze alexithymia in IPVAW perpetrator subtypes. 

With regard to the second objective, we failed to find significant moderators in the association of alexithymia with dropout and reoffending. This does not mean that these variables are unrelated. It should be noted that impulsivity and drug misuse entailed high alexithymia as well as higher rates of dropout and risk of IPVAW recidivism. Moreover, after being included in the model, most of them increased the amount of explained variance. This means that there were not any differences between those individuals who scored high or low in impulsivity and/or drug misuse. Maybe these variables should be considered as complementary to explain these complex phenomena instead of assuming the existence of moderating or mediating relationships. Nevertheless, the absence of significant moderation models also might be explained by methodological questions, such as the methods chosen to assess these variables. In fact, several studies highlight the importance of studying the frequency of use along with accounting for the amount of drug consumption during each use when studying drug misuse effects [34,35,36]. It is also important to employ other ways to assess psychological variables, such as cognitive tests in combination with self-reports [37,38,39].

Despite the promising and interesting results of this study, there are several limitations that reduce the external validity of our results. Firstly, the correlational and non-causal nature of our study limited the causality of the associations between variables. Moreover, the limited sample size based exclusively on the Spanish population also should be considered as an important limitation. In fact, due to the reduced sample size, the possibility of conducting more complex statistical analyses was limited. Moreover, it would be necessary to replicate the results in future studies with a more controlled study and a larger sample size. Secondly, the absence of official reoffending rates certainly decreases the validity of our results. Thus, it would be necessary to obtain this data from legal institutions in order to correlate with data included in this study. Furthermore, the majority of variables included in this study were from self-reports, so it would be necessary to complete our data with objective (i.e., cognitive tests, laboratory tasks, neuroimaging techniques) and qualitative measurements (behavioral control during therapy, personality, and emotional assessment) of alexithymia and impulsivity for future research. 

## 5. Conclusions

Our study reinforces the need to develop good screening methods designed to detect the initial needs of IPVAW perpetrators and, in turn, to prevent violent recidivism in the long term by effectively helping with information processing and behavior regulation. In fact, it is necessary to design a battery of neuropsychological tests, self-reports, and psychophysiological techniques in order to build specific subgroup profiles of IPVAW perpetrators during the initial stages of interventions. This would make it possible to determine the therapeutic needs of each subgroup. In this sense, some individuals might benefit from cognitive-behavioral therapy (CBT) alone and other groups of IPVAW perpetrators might need the combination of this type of intervention together with coadjutant treatments. For example, there is a considerable percentage of IPVAW perpetrators that present cognitive deficits, which tend to be highly correlated with drug misuse [39. In this case, these IPVAW perpetrators need not only CBT but also substance use disorder treatment and cognitive training. The combination of interventions helps reinforce treatment adherence in these individuals [40,41,42,43,44]. Our novel data can help to improve our knowledge of factors involved in IPVAW perpetration and the ways to prevent it. To increase treatment adherence when dealing with alexithymia, it would be interesting that future CBT for IPVAW perpetrators includes self-awareness training, which has a demonstrated efficacy in reducing alexithymic traits [44]. The present study focused on analyzing alexithymia and further studies should consider how it changes during treatment as well as ways to promote it in order to reduce future recidivism. In fact, improvements were reported in several variables, which are closely related to alexithymia, after IPVAW treatment [40,41,42,43,44]. Nonetheless, little is known regarding changes in alexithymia after these treatments. As we gain knowledge analyzing the relationships between previously mentioned factors, it could be applied to develop valid screening methods along with strengthening current rehabilitation programs and designing adjuvant treatments to increase their effectiveness.

## Figures and Tables

**Table 1:** Mean + SD and percentages of socio-demographic and drug use variables of participants.

**IPVAW Perpetrators (*n* = 118)**		
Age (years)		41.47 ± 12.46
Nationality	Spanish Latin Americans	76% 24%
Marital status	Married/Cohabiting Divorced/Single	16.9% 83.1%
Educational level	Primary/lower secondary Upper secondary/vocational training University	56.8% 36.4% 6.8%
Employment status	Employed Unemployed	56% 44%
TAS-20 total score		63.92 ± 16.97
Self-reported impulsivity		32.03 ± 5.90
**Drug Misuse**		
AUDIT_total score (alcohol)_		5.98 ± 6.22
Cannabis	Yes No	32% 68%
Number of joints of cannabis per week		9.93 ± 14.19
Cocaine	Yes No	11% 89%
Number of grams per week		1.27 ± 1.02
**Treatment Accomplishment and Initial Risk of Reoffending**		
Dropout	Yes No	19.5% 80.5%
Number of treatment sessions received		23.42 ± 11.66
SARA (initial stages of treatment)		10.53 ± 6.34

**Table 2:** Correlation coefficients for self-reported impulsivity (dummy coded) and drug misuse classification with dropout and risk of reoffending. * *p* < 0.05, ** *p* < 0.01.

IPVAW Perpetrators (*n* = 118)				
	TAS-20	Dropout	Number of Treatment Sessions Received	SARA
**Impulsivity group**	0.213 *	0.153	−0.226 *	0.259 **
**Alcohol group**	0.320 *	0.229 *	−0.247 **	0.376 **
**Cannabis group**	0.243 *	0.256 *	−0.323 **	0.328 **
**Cocaine group**	0.204 *	0.237 *	−0.162	0.220 *
